# Reduced Capacity for Parafoveal Processing (ReCaPP) Leads to Differences in Prediction Between First and Second Language Readers of English

**DOI:** 10.3390/jemr18020003

**Published:** 2025-02-26

**Authors:** Leigh B. Fernandez, Shanley E. M. Allen

**Affiliations:** Center for Cognitive Science, Psycholinguistics and Language Development Group, University of Kaiserslautern-Landau, 67663 Kaiserslautern, Germany; allen@rptu.de

**Keywords:** eye movements, parafoveal processing, individual differences, bilingualism

## Abstract

Research has shown that first (L1) and second language (L2) speakers actively make predictions about upcoming linguistic information, though L2 speakers are less efficient. While prediction mechanisms are assumed to be qualitatively the same, quantitative prediction-driven processing differences may be modulated by individual differences We tested whether L2 proficiency and quality of lexical representation (QLR) impact the capacity of L2 readers to extract parafoveal information while reading, leading to quantitative differences in prediction. Using the same items as Slattery and Yates, we investigated the impact of predictability and length of a critical word on bottom-up parafoveal processing, measured by skipping rates, and top-down predictability processing, measured by reading times. Comparing our L2 English to their L1 English data, we found that L2 speakers skipped less and had longer gaze duration. However, both groups showed increased skipping rate and decreased gaze duration for predictable relative to unpredictable words and for shorter relative to longer words. We argue that L1 and L2 predictability mechanisms are qualitatively the same and quantitative differences stem from L2 speakers’ Reduced Capacity for Parafoveal Processing, the ReCaPP hypothesis.

## 1. Introduction

An increasing amount of research shows that first language speakers (L1) can make use of multiple sources of information to predict upcoming linguistic content before encountering it (for reviews see [1,2,3,4,5]). Although there is by now a large number of studies on prediction building during sentence processing, the bulk of what we know comes from research with L1 speakers. Much less research has focused on L2 speakers, despite the fact that bilinguals make up the majority of the world’s population [6].

In its early stages, research investigating second language (L2) prediction focused primarily on whether L1 and L2 speakers employ qualitatively different prediction mechanisms [7,8,9]. However, after substantial research showing that L2 speakers can indeed make predictions in many contexts (see reviews in [10,11]), the focus has changed to investigating the quantitative differences between L1 and L2 predictions (e.g., due to individual differences), assuming that the prediction mechanisms between L1 and L2 speakers are fundamentally the same (see [10]). Kaan [12] proposes several individual difference factors that may underlie prediction-driven processing differences in L2 speakers (e.g., strength of stored frequency information, experience, competing lexical information, quality of lexical representation, processing strategies). However, the why, how, and when remain unclear (e.g., [12]).

In the present paper, we thus explore in detail one potential explanation for why, how, and when these individual difference factors lead to less possibility for L2 speakers to make use of prediction mechanisms while reading and thus result in reduced prediction during L2 processing. In particular, we point to a Reduced Capacity for Parafoveal Processing, i.e., the ReCaPP hypothesis, as a key possible explanation for these effects, arguing that reduced prediction stems from individual differences that directly impact the ability of L2 readers to extract information from the parafoveal area. To provide evidence for this hypothesis, we conduct a conceptual replication with L2 speakers of previous research that investigated the impact of parafoveal processing on predictive reading behavior in L1 speakers [13], while exploring two individual difference factors, language proficiency and quality of lexical representation. We also conduct a direct comparison between L2 and L1 data to further support our claims. We believe that focusing on parafoveal processing will provide a more comprehensive understanding of why, how, and when quantitative prediction differences arise between L1 and L2 readers and will provide a more comprehensive understanding of how individual differences impact the mechanisms that drive L2 processing.

### 1.1. Parafoveal Processing During L1 and L2 Reading

When the eye fixates on a word during reading, it is primarily ascertaining information about the word itself in the fovea, the center of the retina, which has the highest visual acuity (approximately 2 degrees of visual angle from the fixation). However, it is also ascertaining information about words in the parafovea, the area outside the fovea (extending to approximately 5 degrees of visual angle, e.g., Schotter et al. [14]). The parafoveal area is important as it allows the readers to preprocess upcoming information that is not being directly fixated upon and allows readers to plan upcoming eye movements. Research on parafoveal processing has found that L1 speakers are able to extract orthographic, phonological, morphological, and under certain circumstances, semantic information from the parafoveal area (see [14] for a review). It has also been found that for L1 speakers, when a foveal word is more difficult to process, there are fewer resources available to invest in parafoveal processing [15].

Parafoveal processing is an important contributor to reading because, among other things, it enables readers to make use of the predictability information of the upcoming word(s). Readers use bottom-up parafoveal information in tandem with top-down predictability information in order to achieve a predictability benefit during reading. When a word is highly predictable, it is often read faster or even skipped during reading, compared to a less predictable word (e.g., [16,17,18]). This is at least partly because more predictable words are more likely to be processed in the parafovea before being fixated. It is generally assumed that if a word is skipped, it must have at least in part been processed parafoveally (e.g., [14]), but even if a word is not skipped, readers can obtain useful information parafoveally that can lead to shorter fixations when the word is fixated upon. Thus, word skipping and reading speed are two key indicators of the importance of parafoveal processing to prediction.

Evidence for this derives from a number of studies using the gaze-contingent boundary paradigm (GCB), in which a word or word-like string of letters is presented in the parafoveal area and then changed to a target word once the eye gaze crosses a boundary. For instance, using the GCB, Balota et al. [19] found that readers were more likely to skip a critical word when it was masked with a predictable word based on context, or a visually similar mask to the predictable critical word, relative to when the target word was masked with a less predictable, a visually dissimilar, or an anomalous mask. This suggests that predictability-based skipping occurs only when the parafoveal information is similar to the predicted word. Additionally, Balota et al. [19] found longer reading times on predictable target words compared to unpredictable target words when an anomalous word was viewed parafoveally. This suggests that if readers do not have valid parafoveal information, reading time will increase more for predictable words than for unpredictable words (see also [20,21,22,23,24]). Staub [5] also used evidence from word skipping to show that predictability effects during reading are at least partially established parafoveally. He argued that the parser triggers a graded activation of possible upcoming words during reading, rather than a single word, which in turn facilitates parafoveal processing of early lexical or pre-lexical information such as visual features and orthography. Similarly to Balota et al. [19], he argued that predictability-based skipping only occurs when the parafoveal information closely (or completely) matches the critical word. Together, this research suggests that the facilitated skipping and the faster reading times that predictability affords are contingent on extracting valid parafoveal information during reading.

In addition to word similarity, word length is also processed parafoveally and contributes to skipping during L1 reading. In a seminal study, Rayner et al. [18] manipulated word length with short (4–6 letters), medium (7–9 letters), and long (10–12 letters) words, as well as predictability (based on sentence context). They found that shorter words were skipped more than longer words, and predictable words were skipped more than unpredictable words, but there was no interaction between length and predictability. This suggests that parafoveal word length information does not constrain lexical candidates, and therefore word length information and lexical information exert independent effects on word skipping (however, see [23,25] for evidence of word length and predictability interaction when manipulating parafoveal previews).

Importantly, individual differences play a key role in the way that parafoveal processing interacts with predictability. In a recent replication of Rayner et al. [18] using the same materials, Slattery and Yates [13] again found that shorter and more predictable words were more likely to be skipped in L1 readers, with no interaction; in other words, that parafoveal processing facilitated using predictability information. However, they also found that two measures of individual differences modulated this effect: quality of lexical representation (QLR, via spelling recognition and spelling to dictation) and reading skill. Perfetti and Hart [26] argued that high QLR (i.e., knowing a word’s orthographic, phonological, semantic, and syntactic qualities) leads to easier identification of words during reading. Relatedly, skipping rate has been found to be positively correlated with print exposure [27], and it has been argued that increased knowledge and experience may lead to increased skipping rates [28]. Additionally, it has been found that higher reading skill sharpens lexical representations, which leads to quicker lexical access and ultimately enables prediction (e.g., [29,30]). Together this suggests that individuals with higher reading and spelling skills are more efficient at extracting parafoveal information during reading, which facilitates prediction. Slattery and Yates [13] found that QLR impacted skipping rate (skipping likelihood increased as QLR increased) but not gaze duration, while reading skill impacted gaze duration (duration decreased as reading score increased) but not skipping rate. This suggests that individual differences indeed affect the ability to process parafoveally, and should thus crucially be taken into account in future studies.

The research just reviewed makes it clear that L1 speakers use parafoveal information during reading in order to facilitate more efficient use of predictability information, thus leading to faster reading. When it comes to L2 speakers, the picture is not as clear. Given that L2 processing is more cognitively demanding than L1 processing (e.g., [12,31]), it stands to reason that L2 speakers have fewer resources to invest in parafoveal processing. This is important, because, as mentioned previously, when a word is more difficult to process, there are fewer resources available to invest in parafoveal processing [15] so L2 speakers may be particularly impacted when it comes to parafoveal processing.

However, we know little about how this affects prediction during reading because research on L2 reading has tended to examine predictability separately from parafoveal processing. Research directly investigating predictability in L2 speakers during reading typically uses event related potentials (ERPs) during word-by-word reading (e.g., [9,32,33]). However, the method of word-by-word presentation necessarily means that no parafoveal information is available to the readers. Similarly, research investigating parafoveal processing with L2 readers typically does not address its relationship to predictability. Since the latter work is most informative for the present study, we briefly review the relevant L2 research involving parafoveal processing while reading sentences, paragraphs, and extended text (novels).

To the knowledge of the authors, only a handful of published studies investigated parafoveal processing with L2 speakers (e.g., [34,35,36,37,38,39,40,41]). The majority of these studies have investigated semantic parafoveal processing (as opposed to orthographic, phonological, or morphological parafoveal processing), coming to somewhat mixed results: some research found evidence for parafoveal semantic processing [41], some found evidence against parafoveal semantic processing [34,36,37,40], and one study was somewhat inconclusive [35]. Despite these diverse findings about L2 semantic parafoveal processing, all of the previous studies clearly show that L2 speakers are able to extract visual-level features from the parafoveal area.

Word skipping and reading time, two main indicators of parafoveal processing, are also affected when reading in an L2, though little information is available about how this relates to predictability or is affected by individual differences. At least two studies have found that L2 speakers skip less and have longer reading times when reading in their L2 as compared to their L1 [42,43]. During the reading of a novel, Cop et al. [42] found that bilingual speakers skipped approximately 5% less and fixated 9% longer (the average of all fixation durations in a sentence) when reading in their L2 compared to their L1. However, Cop et al. [42] did not investigate predictability in their study, and while their analysis included a predictor of L2 proficiency, they did not include its interaction with any of the independent variables. During paragraph reading, Whitford and Titone [43] found that both older and younger adult bilingual readers skipped less and had longer gaze durations in their L2 compared to their L1. Additionally, there was an interaction between the language of the paragraph (L1 or L2) and the predictability of the paragraph content (using a cumulative Cloze task); however, the authors noted that the paragraphs did not offer much contextual constraint and contained very few highly predictable words, and upon removing those few predictable words from the analysis, the interaction was no longer significant. Additionally, while Whitford and Titone [43] included both the amount of L2 exposure (as a measure of QLR) and its interaction with the independent variables as predictors in their model (with more L2 exposure facilitating L2 word processing while impeding L1 word processing, but only for younger adults), they did not include a measure of L2 proficiency.

It is clear from the two studies just mentioned that L2 speakers skipped less and had increased reading time in general. However, both these studies used naturalistic reading—a method that has high ecological validity but unfortunately does not allow for careful manipulation of items that directly test the relationship between prediction and reading behavior. Therefore, in the current study, we examine the role of parafoveal processing in prediction during L2 reading using the materials from Rayner et al. [18] and Slattery and Yates [13]. These materials are specifically designed to systematically investigate the effects of predictability and length on reading behavior, with particular attention to word skipping and reading speed as dependent variables providing insight into the role of parafoveal processing. Further, we examine the effect of two individual difference variables that have been shown to affect parafoveal processing in previous research: proficiency and QLR.

### 1.2. Individual Differences

Given the importance of individual differences in both L1 and L2 processing, in the current study, we examine both proficiency and quality of lexical representation (QLR). Proficiency has been shown to have mixed effects on prediction and parafoveal processing (as measured by skipping and gaze duration). For prediction, some research has found that L2 speakers show L1-like predictive abilities at higher proficiency levels (e.g., [44,45,46]). However, not all research has found such a relationship between proficiency and predictive abilities [10,47,48,49,50,51,52], suggesting that predictive abilities do not come part and parcel with higher proficiency. In terms of parafoveal processing, Wang et al. [40] found that L2 speakers with higher proficiency were more efficient at extracting parafoveal information, but they found no impact of proficiency on skipping (note, however, that the authors designed their items to have low predictability in order to discourage readers from skipping the target word). In two recent studies, Fernandez et al. [35,36] measured proficiency using the Oxford Placement Test—Part A (the same test employed in the current study) and found that skipping rates (and thus presumably parafoveal processing) increased with proficiency for L2 speakers of English. However, this was only the case in the study that investigated predictable target words in a sentence [35], and not in a somewhat similar study that investigated unpredictable target words in a sentence [36]. Additionally, neither study found a relationship between proficiency and gaze duration. Given that studies have not consistently shown a relationship between proficiency and reading behavior (even when using the same proficiency test), we include a measure of proficiency in the current study in hopes of shedding light on this discrepancy, with items that directly manipulate predictability.

As for QLR, previous research showed that a higher QLR makes the identification of words during reading easier, leading to the assumption that readers with a higher QLR are more efficient at extracting information from the parafoveal area (e.g., [53]). For L1 speakers, Slattery and Yates [2] found that greater QLR (measured via spelling recognition and spelling to dictation) led to more skipping but did not impact gaze duration. For L2 speakers, Whitford and Titone [43] found that greater QLR (measured via L2 exposure) facilitated parafoveal processing (at least for younger adults). Additionally, Fernandez et al. [35] found that greater QLR (measured via spelling recognition) led to more skipping. Given the importance of QLR in the processing of parafoveal information in both L1 and L2 speakers, we include a measure of QLR via a spelling recognition test in the current study.

### 1.3. Current Study

Research on eye movements while reading has found that L1 speakers use top-down predictability information in tandem with bottom-up visual parafoveal information during reading, highlighting the importance of parafoveal processing in establishing a predictability benefit. Research with L2 speakers suggests that not only do L2 speakers use parafoveal information less efficiently than L1 speakers, but they also make predictions less efficiently. Therefore, in the current study, we investigate whether differences between L1 and L2 predictability processing stem from a Reduced Capacity for Parafoveal Processing, the ReCaPP hypothesis, rather than qualitatively different prediction mechanisms. Given that L2 speakers must invest more cognitive resources in L2 processing, we hypothesize that they have fewer resources available to process parafoveal information, which causes them to skip less during reading and to have longer reading times. Additionally, we hypothesize that their capacity to process parafoveal information will further be modulated by individual differences. For maximum comparability to previous work, we use the materials from Rayner et al. [18] and Slattery and Yates [13], which include both high- and low-predictability sentences while varying the length of the critical word. To test the influence of individual differences, we included a measure of QLR (a spelling recognition test) and a measure of English proficiency (Oxford Placement Test—Part A). In addition, we directly compare the L1 speakers in the Slattery and Yates [2] paper to the L2 speakers in the current study.

If L2 speakers employ the same prediction mechanisms as L1 speakers, we hypothesize that both groups will show similar patterns in their use of both bottom-up parafoveal information and top-down predictability information during reading. Similarly to previous research [2], we measure bottom-up parafoveal processing via skipping rate and top-down predictability processing via gaze duration. If readers can obtain useful information parafoveally, this should lead to higher skipping rates and shorter fixations if the word is fixated upon. Therefore, we hypothesize that skipping rate will decrease, and gaze duration will increase for unpredictable relative to predictable words, and that skipping rate will decrease, and gaze duration will increase as word length increases, with no interaction. Given that individual differences directly impact the ability to extract information from the parafoveal area, we believe that L2 speakers’ capacity to use parafoveal information, and in turn predictability information, will be modulated by QLR and potentially proficiency. We hypothesize that L2 skipping rate will increase and gaze duration will decrease as the QLR score increases, given that increased QLR should lead to sharper lexical representations, which should in turn aid in lexical identification during reading. Given that the role of proficiency is not consistent across studies (even across studies using the same proficiency test), we put forward no hypothesis about the effect of proficiency on gaze duration and skipping during L2 reading but expect that our analysis can shed some light on this question. If differences do arise between the L1 and L2 groups, we hypothesize that they will be quantitative in nature, such that the patterns are the same but L2 speakers may skip less overall and have longer gaze durations.

## 2. Materials and Methods

### 2.1. Participants

A total of 75 participants were recruited from the University of Kaiserslautern-Landau. Five participants were removed due to a chin rest malfunction; participants slowly drifted down, leading to decreasing accuracy as the study progressed (the chin rest was then replaced). Therefore, 70 participants were included in the analysis. All participants were L1 speakers of German with L2 English and had not learned a second language before age 6. See Table 1 for additional information.

### 2.2. Materials

Experimental materials were taken from Rayner et al. [17]. The experiment consisted of 59 trials: 5 practice trials and 54 critical items. The critical items consisted of 54 sentence pairs, each comprising two sentences (see Table 2). The first sentence varied across the sentence pairs and set up the predictability of the critical word in the second sentence. The second sentence was the same across sentence pairs and contained the critical word. The critical words (underlined in Table 2) were manipulated to be short (4–6 letters), medium (7–9 letters), or long (10–12 letters). Therefore, two experimental versions were created with length and predictability rotated across the versions.

For the English proficiency test (Oxford Placement Test—Part A), participants selected a sentence continuation from three options. The test consisted of 50 items, and the score was converted into a percentage. For the misspelling identification task (to measure the quality of lexical representation (QLR), participants identified any misspelled word from a list of 215 words (of which 51 words were misspelled). The score was calculated as a percentage corrected for the unequal proportion of correctly spelled words and incorrectly spelled words by averaging the proportion of correct answers in both types of words (see Table 1).

### 2.3. Apparatus

Stimulus presentation was programmed using Experiment Builder software (Version 2.1.140, SR Research, Kanata, ON, Canada), and eye movements were recorded with an Eyelink 1000 (SR Research, Kanata, ON, Canada) desktop mount sampling at 1000 Hz. Viewing was binocular, but only the right eye was tracked. Participants sat approximately 70 cm away from the screen, and a chin rest was used to minimize head movements. Stimuli were presented on a Samsung (Seoul, Republic of Korea) SyncMaster 959NF 19″ flat screen cathode ray tube (1024 × 768 resolution; 120 Hz refresh rate), and approximately 3 characters subtended 1° of visual angle. Items were presented in black on a gray background in Courier New with a font size of 15.

### 2.4. Procedure

Participants first read and signed an informed consent form. They then completed the eye-tracking part of the study. Participants were calibrated on a 9-point calibration screen and were instructed to read silently and to answer a yes/no comprehension question that probed the interpretation of the sentence they had just read. Comprehension questions occurred after 1/3 of the trials and were answered by pressing “z” for yes and “m” for no on a standard English keyboard. The study was self-paced such that participants could take a break as needed (between trials). Recalibration took place obligatorily halfway through the study, as well as after any breaks and as otherwise needed. Following the eye-tracking task, participants completed a language history questionnaire and two individual differences measures. First, they completed an English proficiency test (Oxford Placement Test—Part A). Second, participants completed a misspelling identification task to measure their quality of lexical representation (QLR). The study took approximately 60 min, and participants were paid EUR 8 or received course credit for their participation.

### 2.5. Analysis

Prior to analysis, trials were eliminated for one of three reasons: (1) if a blink occurred on the fixation immediately before or after the critical word (6.26% of trials); (2) if the launch site of the first pass saccade could not be calculated (e.g., if the saccade occurred outside of an interest area; 1.53% of trials); and (3) if the launch site of the first pass saccade occurred from more than 15 characters away (given that the items occurred over more than one line, we assumed that trials with large launch sites were not representative of eye movement behavior of processing a critical word; 9.12% of trials). Comprehension accuracy was relatively high (mean = 88.19%, range = 61–100%). We additionally ran all the analyses with the two participants who scored below 70% removed. There was only one minor difference, as noted in the results section; therefore, we kept all 70 participants in the overall analyses.

Our main dependent variables, as in Slattery and Yates [2], are gaze duration (the duration of all first past fixations on the critical word before the eye moves progressively or regressively) and skipping rate (proportion of critical words that were skipped during first pass reading). We believe both measures are of equal importance and will inform us about top-down predictability (gaze duration) and bottom-up parafoveal (skipping rate) processing of predictability and length information during reading. We first analyze the gaze duration and skipping rate for L2 data independently and then we directly compare the L2 data to the Slattery and Yates [2] L1 data. The data from the Slattery and Yates [2] paper was obtained from the authors and is used for quantitative comparison between L1 (their data) and L2 (the current data) speakers.

For our L2 analyses, given the inherent skew that comes with duration measures, we opted to run a slightly different model than in the original Slattery and Yates [2] paper for the gaze duration measure. Instead of transforming the data (which can lead to spurious results [54]), we used the raw gaze duration data and accommodated the shape of the skewed data in the model. Gaze duration was analyzed with generalized linear mixed-effects models (GLMM) specified with an identity link (specifying a linear relationship between predictors and observed responses) and a Gamma distribution (specifying duration is positive; see Lo and Andrews [54]). In line with Slattery and Yates [2], skipping rate was analyzed using a mixed logit model with GLMM using the lme4 package [55] in R [56]. Results include *p*-value estimates from the lmerTest package [57]. In line with Slattery and Yates [2], we ran two models: the first included the fixed effects of word length (centered) and predictability (sum contrast coded 0.5/−0.5) and their interaction. The second model was built by adding the interaction of proficiency and QLR (Note: proficiency and QLR have a 0.62 correlation) to the fixed effects and random effects of the first model.

To test differences between L1 and L2 readers, we directly compared the current L2 data with the L1 skipping data from the Slattery and Yates [2] paper. Given that this comparison was performed post hoc, we did not have a priori hypotheses and thus consider this an exploratory analysis. Therefore, we apply a Bonferroni correction based on the four L2 models that we have run, in an aim to decrease the likelihood of Type II error, giving us an alpha threshold of 0.013 (0.05/4; [58]). Similarly to the above, our model comparing L1 and L2 speakers included the fixed effects of word length (centered) and predictability (sum contrast coded 0.5/−0.5). We also included the main effect of language (sum contrast coded 0.5/−0.5) and their interaction. Given that the battery of individual difference tests was not the same across the two studies, we do not include them in our model. Therefore, we present 6 models below: 3 gaze duration models (L2 without individual differences, L2 with individual differences, and L1 compared to L2) and 3 skipping rate models (L2 without individual differences, L2 with individual differences, and L1 compared to L2).

## 3. Results

The L1 and L2 descriptive statistics are reported in Table 3 (see https://osf.io/af5q3/ (accessed on 12 January 2025) for L2 data and statistical code).

### 3.1. Gaze Duration

#### 3.1.1. L2 Data—Gaze Duration Model 1

In our first model, we compared gaze duration for L2 speakers, including the fixed effects of word length (centered) and predictability (see Table 4 for model output information). The random effects structure was maximally specified [59]. Gaze duration increased as word length increased (t = 7.10, *p* < 0.001). There were longer gaze durations for unpredictable relative to predictable words (t = −2.25, *p* = 0.025). There was an interaction of word length and predictability (t = −2.23, *p* = 0.026), with a greater increase in gaze duration for unpredictable words as length increased relative to predictable words.

#### 3.1.2. L2 Data—Gaze Duration Model 2

**Figure 1:**
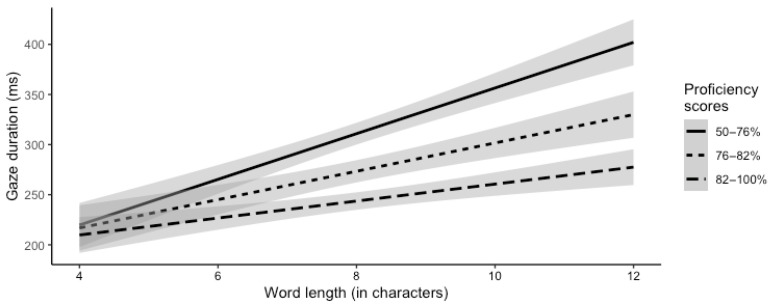
Gaze duration across word length broken down by proficiency score (gray shading indicates a 95% confidence interval).

The second model was built by adding the interaction of the two centered individual differences measures, proficiency and QLR, to the fixed effects and random effects of the first model (see Table 5 for model output information). The random effects structure was maximally specified. The main effects of word length and predictability and their interaction were the same as in the previous model. Gaze duration increased as proficiency score decreased (t = −6.69, *p* < 0.001) and increased as QLR score decreased (t = −2.22, *p* = 0.026). There was a two-way interaction between word length and proficiency (t = −2.04, *p* = 0.040). In the model, proficiency was a continuous variable, but for visualization of the interaction, we split the variable into three levels (see Figure 1). Gaze duration was similar for short words regardless of proficiency. However, as word length increased, proficiency impacted gaze duration differentially. There was a graded effect: gaze duration increased as word length increased, and this increase was greater as proficiency decreased.

**Figure 2:**
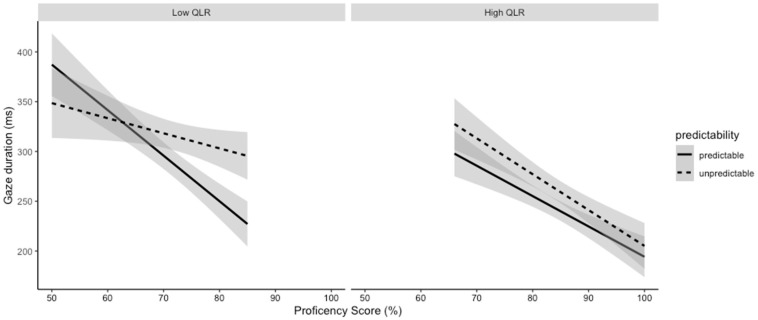
Gaze duration of predictable and unpredictable words across proficiency, broken down by QLR score (gray shading indicates a 95% confidence interval).

Additionally, there was a three-way interaction between predictability, QLR, and proficiency (t = 2.27, *p* = 0.023). Again, to visualize the interaction, we subset the QLR score into two levels, each with 50% of the participants: scorers between 50 and 76% (n = 35) were in the low QLR group, and scorers between 76 and 94% (n = 35) were in the high QLR group (see Figure 2). We found that low QLR readers with low proficiency had the longest gaze durations and showed no impact on predictability. However, as proficiency increased low QLR readers showed a predictability effect, with predictable words having shorter gaze durations than unpredictable words. For the high QLR group, overall, gaze duration decreased with proficiency and there was no impact of predictability on gaze duration. However, differences in gaze duration between predictable and unpredictable items did visually decrease as proficiency increased.

#### 3.1.3. L1 and L2 Data—Gaze Duration Model 3

In our third model we compared gaze duration for both L1 (taken from Slattery and Yates [13]) and L2 speakers including the fixed effects of word length (centered), predictability, and Language (see Table 6 for model output information). The random effects structure was maximally specified. Gaze duration increased as word length increased (t = 7.21, *p* < 0.001), gaze duration decreased for predictable relative to unpredictable words (t = −4.37, *p* < 0.001), and there was an interaction between word length and predictability (t = −2.66, *p* = 0.007), with length impacting unpredictable items more than predictable items. Gaze duration was shorter for L1 speakers relative to L2 speakers (t = −3.30, *p* < 0.001). There was an interaction between word length and language (t = −4.98, *p* < 0.001), with both groups showing similar gaze durations for short words, but L2 speakers showing a greater increase in gaze duration as word length increased relative to L1 speakers. Nothing else reached significance.

### 3.2. Skipping Rate

#### 3.2.1. L2 Data—Skipping Rate Model 1

The first skipping model included the same fixed and random effects used in the gaze duration model with the addition of the main effect of the centered launch site (see Table 7 for model output information). The random effects structure was maximally specified. Skipping rate decreased as word length increased (z = −6.04, *p* < 0.001). Skipping rate also decreased for unpredictable words relative to predictable words (z = 1.94, *p* = 0.052). Upon removing the two participants with an accuracy of less than 70%, the main effect of predictability became marginally significant (*p* = 0.070). However, the main effect remained when the individual differences were included. Finally, skipping rate decreased as the launch site increased (z = −13.94, *p* < 0.001). Nothing else reached significance.

#### 3.2.2. L2 Data—Skipping Rate Model 2

For the second model, centered QLR and proficiency were added to the fixed and random effects of the first model (see Table 8 for model output information). The random effects structure was maximally specified (Note: Slattery and Yates’s [13] full model did not converge, so they reduced their fixed effects to include all main effects and two interactions of a priori interest and ran an additional critical word saccade length model. Given that our full model converged, we did not run any additional models). The main effects were similar to the first model in terms of word length, predictability, and launch site. Skipping rate also increased as proficiency increased (z = 1.93, *p* = 0.054). Additionally, there was a three-way interaction between predictability, QLR, and proficiency (z = −3.05, *p* = 0.002). Nothing else reached significance.

**Figure 3:**
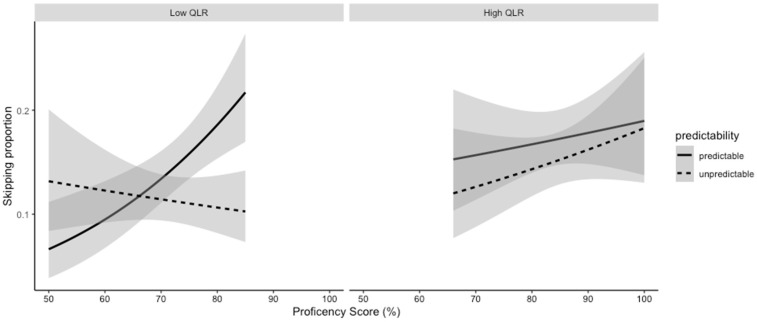
Skipping proportion of predictable and unpredictable words across proficiency, broken down by QLR score (gray shading indicates a 95% confidence interval).

The interaction was broken down in the same way as the gaze duration interaction (see Figure 3). We found that predictability had no impact on skipping for low QLR readers with low proficiency. As proficiency increased, however, low QLR readers showed a predictability effect, with predictable words being skipped more than unpredictable words (and with skipping for unpredictable words decreasing with proficiency). For the high QLR group, overall, skipping increased with proficiency, and there was no impact on predictability. However, differences in skipping between predictable and unpredictable items did visually decrease as proficiency increased.

#### 3.2.3. L1 and L2 Data—Skipping Rate Model 3

In our third model, we compared skipping rate for both L1 (taken from Slattery and Yates [13]) and L2 speakers including the fixed effects of word length (centered), predictability, and language (see Table 9 for model output information). The random effects structure was maximally specified. Skipping rate decreased as word length increased (z = −8.91, *p* < 0.001), and skipping rate increased for predictable relative to unpredictable words (z = 3.01, *p* = 0.002). Skipping rate was greater for L1 readers relative to L2 readers (z = 3.70, *p* < 0.001). There was an interaction between word length and language (z = 2.78, *p* = 0.005), with both groups showing similar skipping rates for short words, but L2 speakers showed a greater decrease in skipping as word length increased. Nothing else reached significance.

## 4. Discussion

It is well established that L1 speakers make predictions about linguistic information. When it comes to L2 speakers, however, early studies failed to find evidence of predictive processing by L2 speakers, leading some researchers to argue that L2 predictive mechanisms differed qualitatively from L1 predictive mechanisms (e.g., [7,8,9]). However, the more recent consensus is that L2 speakers are indeed able to make predictions, and that predictive mechanisms themselves do not differ between L1 and L2 speakers [10,11]. This has led to a shift in L2 research, away from testing whether L2 prediction mechanisms differ qualitatively from L1 prediction mechanisms, and rather focusing on the individual difference factors that modulate quantitative prediction-driven differences [12,13]. It is clear that prediction is at least in part established parafoveally (e.g., [5]) and that this ability is modulated by individual differences (e.g., [35,36,40,53]). However, little is understood about exactly how, why, and when these individual differences impact L2 prediction. In the present study, we investigated whether these individual difference factors crucially lead to a Reduced Capacity for Parafoveal Processing in L2 speakers, the ReCaPP hypothesis, and thus to less efficient use of parafoveal information in accessing predictability information. We further tested this using two individual difference factors that have been shown to impact the ability to extract parafoveal information: L2 QLR and L2 proficiency.

As in Slattery and Yates [13], we compared skipping rate and gaze duration on a target word, since these measures provide important information on top-down predictability (gaze duration) and bottom-up parafoveal (skipping rate) processing during reading. Overall, we found that L2 speakers skipped less and had greater gaze durations relative to L1 speakers (in line with [42,43], who both used free-form texts). However, we found for both groups that skipping decreased and gaze duration increased as word length increased. This is one of the most robust findings in L1 skipping literature [2,17,58,59], and we have now found this using controlled materials with L2 speakers as well. We also found that both groups were more likely to skip and to have shorter gaze durations for predictable relative to unpredictable words (cf. [13,18,19,21]). This is the first study to show this pattern of results with L2 speakers using items specifically designed to test the impact of both prediction and word length during sentence reading. Interestingly, we found that language and predictability did not interact, suggesting that even though L2 speakers read more slowly than L1 speakers overall, L1 and L2 speakers process predictability similarly. Overall, these findings suggest that L1 and L2 speakers’ predictive mechanisms are qualitatively the same, but that quantitative predictability differences arise because L2 speakers have reduced capacity for parafoveal processing, which leads to an overall reduction in skipping and an increase in reading times. However, as discussed below, we found that predictability was modulated by a combination of individual differences.

In terms of individual differences, we explored the role of QLR and L2 language proficiency. Unexpectedly, we did not find a main effect of QLR on its own on skipping rate (although, as described below, it does interact with other factors), even though it has previously been shown to play a role in both L1 ([13,53]) and L2 ([35,43]) parafoveal processing and skipping behavior. However, we did find a main effect of QLR on gaze duration, with gaze duration decreasing as QLR increased. Notably, each of the studies mentioned here has used a different proxy for measuring QLR, and we recommend future research using a validated QLR test (e.g., [59]). In terms of L2 language proficiency on its own, its relationship to prediction is somewhat unclear, since better prediction abilities do not necessarily come with higher language proficiency (e.g., [10,48,49,50,51,52]). In the present study, however, we found an impact of proficiency on both gaze duration (decreasing with higher proficiency) and skipping rate (increasing with higher proficiency). We also found an interaction between word length and proficiency, with length effects increasing with decreasing proficiency. In sum, our study suggests that both individual differences impact L2 parafoveal processing.

We also found a three-way interaction between proficiency, QLR, and prediction for both gaze duration and skipping. We suggest that as readers with lower QLR scores become more proficient in their L2, their reading strategy shifts, and they begin to rely more on top-down contextual information. It is clear that both QLR and proficiency play an important role in L2 parafoveal processing, and that neither measure can fully capture the complex patterns that emerge. This joint influence suggests that top-down predictability effects in L2 reading are modulated by a combination of orthographic precision of lexical representations (as measured by spelling skill) and proficiency, leading to more efficient bottom-up parafoveal processing. This may explain why previous research investigating proficiency during reading, and also predictability and proficiency, has not shown consistent findings (e.g., [35,36,40]). For example, in both Fernandez et al. studies [35,36] (that used the same proficiency test used in the current study), QLR and proficiency were examined independently, but not jointly. We could not directly compare the role of L1 and L2 individual differences on top-down predictability and bottom-up parafoveal processing due to different measures being used across the studies. However, we encourage future research across L1 and L2 speaker groups using the same standardized individual difference measures.

As mentioned previously, L2 research is reorienting to identify why, how, and when individual difference factors lead to prediction-driven differences during L2 processing. The findings from the present study support the growing literature that L1 and L2 prediction mechanisms are qualitatively the same and that differences arising between the two groups are quantitative in nature and are modulated by individual differences (e.g., [10,11]). Particularly, we argue that L2 speakers have fewer cognitive resources to invest in parafoveal processing, which leads to quantitative predictability differences between L1 and L2 speakers. However, the ability to use parafoveal information is impacted by individual differences: top-down predictability processing is modulated by both orthographic precision of lexical representations and proficiency, which in turn affects bottom-up parafoveal processing. Given this pattern of results, we put forward the hypothesis that quantitative prediction-driven differences between L1 and L2 speakers during reading stem from L2 speakers’ Reduced Capacity for Parafoveal Processing, or the ReCaPP hypothesis.

Alternatively, it may be that predictability effects appear later for less skilled readers. That is, more skilled readers are able to generate predictions more quickly and/or use fewer resources, which allows them to use parafoveal information more efficiently. Indeed, it has been found that L1 German/L2 English speakers with high English skills have a larger perceptual span when reading in English relative to speakers with lower skill [60], which may lead to earlier predictability effects. Research using a combination of self-paced reading and masked priming (e.g., [61]) may be able to shed further light on the time course of predictability effects and the role of parafoveal in L2 reading. Nevertheless, while the time course of predictability may differ based on skill, we still believe this explanation fits within the ReCaPP hypothesis, in that L1 and L2 prediction differences are quantitative (and driven by individual differences) not qualitative in nature.

Unexpectedly, we found an interaction between word length and predictability for all gaze duration models (but not for skipping). This was unexpected because Slattery and Yates [13] did not find this interaction using the same items and paradigm as the present study (nor did [17]), and because the majority of research with L1 speakers has found additive rather than interactive effects of word length and predictability (e.g., [21,62]). These data suggest that L2 speakers use word length information in combination with top-down contextual information to narrow down lexical candidates during reading. The slower gaze duration for L2 speakers, coupled with restricted parafoveal information relative to L1 speakers, may lead to L2 speakers being able to identify short words in the parafovea but not longer words. This would suggest that while L2 speakers exhibit a ReCaPP, they are still able to identify visual features in the parafovea, which leads to decreased foveal gaze duration. This idea is further supported by the fact that word length interacted with language: both L1 and L2 speakers showed similar gaze durations and skipping rates when words were short, but L2 speakers showed a greater impact of longer words on both measures. In order to shed further light on the interaction between length and predictability, we suggest future research manipulating the parafoveal preview of a target word (e.g., [23,25,63]). Another seemingly promising line of future research may be to investigate whether skipping and reading behavior changes for L2 speakers as a result of the study instructions and comprehension task. Research has found that skipping rate is influenced by the reading task and strategy (i.e., thorough reading, skimming, spell checking, etc. [64]), and skipping rate may be impacted by visual and cognitive resource availability [65]. It may be that L2 speakers adopt different strategies during reading relative to L1 speakers, which may cause increased investment in foveal processing, leading to ReCaPP.

## 5. Conclusions

In this paper, we put forward the hypothesis that quantitative prediction-driven differences during reading stem from L2 speakers’ Reduced Capacity for Parafoveal Processing, the ReCaPP hypothesis. We support this with evidence from a study with L2 speakers showing that L2 speakers skipped less and had longer gaze durations than L1 speakers. Similarly to L1 speakers, however, L2 speakers’ skipping rate increased and gaze duration decreased as word length decreased, and also for predictable relative to unpredictable words. We found complex interactions between predictability, QLR, and proficiency for both skipping and gaze duration, which suggests that predictability differences stem from these joint influences. This pattern also seems to indicate a change in reading strategy for L2 speakers as both QLR and proficiency increase.

Interestingly, we found an interaction between word length and predictability on gaze duration (with and without individual differences, as well as when the L1 data were included in the models). This is the first study not using parafoveal preview manipulation that has shown that readers are able to use length information to narrow down lexical candidates. We encourage future research manipulating parafoveal previews and using standardized individual differences measures to explore these findings more thoroughly. Overall, these data support the growing literature that L1 and L2 speakers employ the same prediction mechanisms during processing, and when differences do arise, they are quantitative in nature and stem from individual differences that lead to a Reduced Capacity for Parafoveal Processing (ReCaPP) rather than from a qualitative difference.

## Figures and Tables

**Table 1:** Participant information.

N	Mean Age in Years (SD)	Mean Age of English Acquisition (SD)	Gender	Mean Proficiency Score (SD)	Mean QLR Score (SD)
70	24.71 (4.07)	9.75 (1.66)	31 female 39 male	77.90/100 (11.12)	77.03/100 (8.38)

**Table 2:** Example stimuli (the critical word is underlined).

	Short Length	Medium Length	Long Length
First sentence			
Predictable	John has been married for many years now.	Britney always drives way too recklessly.	Megan decided one day to stop eating meat.
Unpredictable	John works very hard and is almost always happy.	Britney is in a really bad mood today.	Megan went to grab some lunch with co-workers.
Second (critical) sentence			
Critical	He loves his wife Joanne, and isn’t afraid to show her affection.	She just got into an accident that wrecked her car.	She officially declared herself vegetarian at lunch today.

**Table 3:** Descriptive statistics of the L1 (obtained from Slattery and Yates [13]) and L2 eye movement measures (standard error in parentheses).

Length	Predictability	Gaze Duration (in ms)	Skipping Rate (in Percent)	Gaze Duration (in ms)	Skipping Rate (in Percent)
		L1		L2	
Short	Predictable	236.89 (3.93)	29.31 (1.08)	248.62 (6.76)	22.40 (1.76)
	Unpredictable	245.27 (3.6)	26.59 (1.43)	235.85 (5.05)	22.70 (1.81)
Medium	Predictable	237.07 (3.83)	20.81 (1.6)	255.62 (7.34)	20.66 (1.74)
	Unpredictable	251.93 (4.04)	18.36 (0.98)	280.07 (6.81)	11.52 (1.37)
Long	Predictable	250.01 (3.88)	10.62 (1.36)	293.72 (8.27)	4.23 (0.88)
	Unpredictable	275.74 (4.28)	8.61 (1.55)	343.26 (10.13)	5.33 (0.98)

**Table 4:** L2 Gaze duration model 1 (* indicates an interaction).

Predictor	Estimate	Standard Error	t Value	*p* Value
(Intercept)	271.38	6.28	43.20	<0.001
Word length	14.75	2.08	7.10	<0.001
Predictability	−16.90	7.53	−2.25	0.025
Word length * Predictability	−6.95	3.11	−2.23	0.026

**Table 5:** L2 Gaze duration model 2 (* indicates an interaction).

Predictor	Estimate	Standard Error	t Value	*p* Value
(Intercept)	270.33	3.01	89.72	<0.001
Word length	13.81	1.33	10.36	<0.001
Predictability	−28.17	6.00	−4.69	<0.001
QLR	−1.24	0.56	−2.22	0.026
Proficiency	−2.53	0.38	−6.69	<0.001
Word length * Predictability	−9.09	2.65	−3.43	<0.001
Word length * QLR	−0.43	0.25	−1.72	0.08
Predictability * QLR	0.76	0.87	0.87	0.39
Word length * Proficiency	−0.34	0.17	−2.04	0.040
Predictability * Proficiency	−0.96	0.68	−1.41	0.16
QLR*Proficiency	0.02	0.03	0.66	0.51
Word length * Predictability * QLR	0.02	0.38	0.05	0.96
Word length * Predictability * Proficiency	0.08	0.30	0.27	0.79
Word length * QLR * Proficiency	0.00	0.01	0.24	0.81
Predictability * QLR * Proficiency	0.13	0.06	2.27	0.023
Word length * Predictability * QLR * Proficiency	0.02	0.03	0.79	0.43

**Table 6:** L1 and L2 Gaze duration model 3 (* indicates an interaction).

Predictor	Estimate	Standard Error	t Value	*p* Value
(Intercept)	259.84	3.61	71.97	<0.001
Word length	9.33	1.29	7.21	<0.001
Predictability	−17.44	3.99	−4.37	<0.001
Language	−20.25	6.13	−3.30	<0.001
Word length * Predictability	−4.69	1.76	−2.66	0.007
Word length * Language	−9.80	1.97	−4.98	<0.001
Predictability *	−0.52	8.09	−0.06	0.95
Language				
Word length * Predictability *	3.44	3.57	0.96	0.34
Language				

**Table 7:** L2 skipping model 1 (* indicates an interaction).

Predictor	Estimate	Standard Error	z Value	*p* Value
(Intercept)	−2.82	0.20	−14.02	<0.001
Word length	−0.42	0.07	−6.04	<0.001
Predictability	0.39	0.20	1.94	0.052
Launch site	−0.46	0.03	−13.94	<0.001
Word length * Predictability	0.09	0.09	0.98	0.33

**Table 8:** L2 skipping rate model 2 (* indicates an interaction).

Predictor	Estimate	Standard Error	z Value	*p* Value
(Intercept)	−2.83	0.40	−7.14	<0.001
Word length	−0.43	0.16	−2.73	0.006
Predictability	0.80	0.27	2.94	0.003
QLR	0.02	0.03	0.75	0.45
Proficiency	0.04	0.02	1.93	0.054
Launch Site	−0.51	0.04	−14.04	<0.001
Word length * Predictability	0.17	0.12	1.37	0.17
Word length * QLR	0.00	0.01	0.12	0.91
Predictability * QLR	0.02	0.04	0.43	0.67
Word length * Proficiency	0.00	0.00	0.11	0.91
Predictability * Proficiency	0.02	0.03	0.84	0.40
QLR * Proficiency	0.00	0.00	−1.04	0.30
Word length * Predictability * QLR	0.02	0.02	0.88	0.38
Word length * Predictability * Proficiency	−0.02	0.01	−1.27	0.20
Word length * QLR * Proficiency	0.00	0.00	0.59	0.56
Predictability * QLR * Proficiency	−0.01	0.00	−3.05	0.002
Word length * Predictability * QLR * Proficiency	0.00	0.00	−0.87	0.38

**Table 9:** L1 and L2 skipping rate model 3 (* indicates an interaction).

Predictor	Estimate	Standard Error	z Value	*p* Value
(Intercept)	−2.07	0.11	−19.5	<0.001
Word length	−0.36	0.04	−8.91	<0.001
Predictability	0.31	0.1	3.01	0.002
Language	0.61	0.17	3.7	<0.001
Word length * Predictability	0.04	0.05	0.91	0.36
Word length * Language	0.15	0.05	2.78	0.005
Predictability *	−0.09	0.19	−0.46	0.65
Language				
Word length * Predictability *	−0.06	0.09	−0.7	0.49
Language				

## Data Availability

The original data and statistical code presented in the study are openly available in OSF at https://osf.io/af5q3/ (accessed on 12 January 2025).

## Abbreviations

The following abbreviations are used in this manuscript:

L1	First language speakers
L2	Second language speakers
QLR	Quality of lexical representation
ReCaPP	Reduced capacity for parafoveal processing
GCB	Gaze contingent boundary paradigm
ERP	Event related potentials
Hz	Hertz
GLMM	Generalized linear mixed-effects models
